# Role of Natural Killer T Cells in the Development of Obesity and Insulin Resistance: Insights From Recent Progress

**DOI:** 10.3389/fimmu.2018.01314

**Published:** 2018-06-11

**Authors:** Masashi Satoh, Kazuya Iwabuchi

**Affiliations:** Department of Immunology, Kitasato University School of Medicine, Sagamihara, Japan

**Keywords:** natural killer T cell, cluster of differentiation 1d, adipocyte, lipid, obesity, insulin resistance, adipose tissue inflammation

## Abstract

Natural killer T (NKT) cells play important roles in adipose tissue inflammation, and thus influence the development of diet-induced obesity and insulin resistance. The interactions between cluster of differentiation (CD)1d and NKT T cell receptor are thought to be critical in this process, as demonstrated in two NKT cell-deficient mouse models—systemic CD1d gene knockout (KO) and prototypic Jα18 KO mice. The latter lacks some repertoires besides invariant (i)NKT cells due to manipulation of the Jα18 gene segment; therefore, the role of iNKT vs. variant NKT cells must be reinterpreted considering the availability of new Jα18 KO mice. NKT cells have varied roles in the development of obesity; indeed, studies have reported contradictory results depending on the mouse model, diet, and rearing conditions, all of which could affect the microbiome. In this mini-review, we discuss these points considering recent findings from our laboratory and others as well as the role of NKT cells in the development of obesity and insulin resistance based on data obtained from studies on conditional CD1d1 KO and new Jα18 KO mice generated through gene editing.

## Introduction

### Obesity as a Chronic Inflammatory Disorder

Inflammation in adipose tissue (AT) is induced by hypertrophy of adipocytes that secrete inflammatory cytokines and chemokines (1) and thus recruit various immune cells such as macrophages, T cells [αβ, γδ, regulatory T cells (Tregs), and natural killer T (NKT) cells], B cells, NK cells, and leukocytes that exist in a steady state in immune organs (2, 3). Fat accumulation is a major factor contributing to meta-inflammation and metabolic dysfunction (1, 4). Obesity alters the microenvironment in AT from an anti-inflammatory to a pro-inflammatory state, leading to impaired immune balance (5, 6). Visceral (V)AT in the lean state predominantly contains M2 macrophages, eosinophils, and Tregs that suppress inflammation and maintain insulin sensitivity (7, 8). By contrast, VAT in obese individuals has more M1 macrophages, cluster of differentiation (CD)8^+^ T cells, NK cells, B cells, and neutrophils that enhance inflammation and reduce insulin sensitivity (9–13). Notably, chronic low-grade inflammation accompanied by obesity is implicated in the etiology of lifestyle-related diseases such as atherosclerosis, type 2 diabetes, and various cancers (14).

### NKT Cells

Natural killer T cells are a unique T cell subset that recognize lipid antigen presented by CD1d (15, 16). α-Galactosylceramide (α-GalCer) is a prototypical ligand recognized by invariant (i)NKT cells that harbors an invariant T cell receptor (TCR) α-chain (Vα14-Jα18 in mouse and Vα24-Jα18 in human) (17). Another type of NKT cell known as variant (v)NKT cells express diverse TCRs that are presumed to recognize various lipid antigens including sulfatide (18). Activated NKT cells secrete large amounts of cytokine that modulate immune balance, implying that they can either enhance or suppress inflammatory and immune responses. NKT cells have been reported to exacerbate, protect against, or have no role in the development of obesity through modulation of AT inflammation (19).

Here, we summarize the correlation between the CD1d/NKT cell axis and obesity with a focus on AT inflammation and discuss factors that may contribute to the discrepancies among reports considering recent progress.

## Opposing Functions of NKT Cells in the Development of Obesity

Many studies have examined whether NKT cells play a role in diet-induced obesity (DIO) and have reported variable results.

### NKT Cells as an Aggravator of DIO

Ohmura *et al*. first demonstrated that iNKT cells induce AT inflammation and glucose intolerance in β_2_-microglobulin (β_2_m) knockout (KO) mice fed a high-fat diet (HFD) and treated with the NKT cell stimulator α-GalCer (20). Since β_2_m KO mice also lack CD8^+^ T cells, the role of NKT cells in obesity has been examined using CD1d KO mice fed an HFD. However, two subsequent studies showed that NKT cell deficiency is insufficient to protect against or aggravate DIO (21) and that CD1d is important for the modulation of metabolic functions *via* a non-NKT cell-mediated mechanism (22). By contrast, we showed that CD1d KO mice lacking both iNKT and vNKT cells showed a reduced body weight (BW) gain along with improved AT inflammation and insulin resistance (23). Meanwhile, Jα18 KO mice lacking only iNKT cells demonstrated similar pathology to wild-type (WT) mice, suggesting that vNKT cells may contribute to DIO in the absence of iNKT cells (23). Wu *et al*. reported that iNKT cells responded to lipid excess and produced pro-inflammatory cytokines that promoted AT inflammation and insulin resistance (24).

### iNKT vs. vNKT Cells

We investigated whether iNKT cells (24) or vNKT cells (23) contribute to the exacerbation of DIO, since distinct measures must be taken to control either subset. We speculated that vNKT cells contribute to the development of DIO in the absence of iNKT cells based on the aforementioned results (i.e., no difference in BW between WT and Jα18 KO mice on an HFD) and some additional observations (23): (1) the NK1.1^+^TCRβ^+^ population in AT was activated upon consumption of an HFD and contained more CD8^+^ but fewer CD4^−^CD8^−^ subsets in Jα18 KO (referred hereafter as Jα281 KO) (25) mice, which differed from observations in either WT or CD1d KO mice; (2) WT mice harbored more non-iNKT (=vNKT) cells in AT; and (3) hepatic mononuclear cells from Jα281 KO mice [which are enriched in vNKT cells including CD1d^+^ antigen-presenting cells (APCs)] transferred insulin resistance to CD1d KO hosts.

However, the Jα281 KO strain was shown to exhibit a marked reduction in TCR diversity, which could affect immune responses (26). Four novel Jα18 KO mouse strains were independently generated after the report (26) by deleting only the *T-cell receptor alpha joining* (*Traj*)*18* locus and leaving the remaining Jα repertoire unperturbed using novel technologies [clustered regularly interspaced short palindromic repeats (CRISPR)/CRISPR-associated protein-9 nuclease or transcription activator-like effector nuclease] (27–30). New Traj18 KO (referred hereafter as simply Traj18 KO) mice gained less weight and had heightened sensitivity to insulin compared with WT mice, suggesting that iNKT cells play a pathogenic role in DIO (30). In that study, the mice were fed the same HFD (HFD-32; CLEA Japan, Tokyo, Japan) as those in our experiments, and Jα281 KO mice fed this diet showed similar BW gain to WT mice. The interpretation of the results from Traj18 KO mice was that iNKT but not vNKT cells exacerbate the development of DIO. Experiments using Vα14-Jα18 transgenic mice lacking low-density lipoprotein receptor also demonstrated that the abundance of iNKT cells increased adiposity by inducing metabolic abnormalities and AT inflammation (31). The DIO results from Traj18 KO mice also imply that reduced TCR diversity or the lack of particular T cell subsets in Jα281 KO but not Traj18 KO mice account for the discrepancy among reports on the involvement of iNKT vs. vNKT cells. Mucosal-associated invariant T (MAIT) cells that utilize Jα33 may be lost in Jα281 KO mice and may thus affect the development of obesity, as was suggested in studies of human subjects (32, 33). However, the actual role of MAIT cells in obesity and their involvement (or that of other T cell subsets) in DIO in Jα281 KO mice require further investigation.

### Protective Role of NKT Cells Against Obesity

Some studies have reported that NKT cells play a protective role against obesity. Regulatory cytokines such as interleukin (IL)-4 and -10 produced by AT iNKT cells prevented the development of DIO (34, 35) and insulin resistance even in mice fed a low-fat diet (36). IL-13-producing innate immune cells such as type 2 innate lymphoid cells (ILC2s), iNKT cells, and vNKT cells were shown to prevent DIO by suppressing inflammation in AT (37). AT-resident iNKT cells express transcriptional repressor E4-binding protein (E4BP) 4 (also known as nuclear factor, IL-3-regulated) but not promyelocytic leukemia zinc finger protein (PLZF), unlike iNKT cells in other tissues, reflecting their anti-inflammatory phenotype (38); moreover, IL-10-producing iNKT cells (NKT10) are enriched in subcutaneous white (W)AT (39). Interestingly, an F108Y substitution in TCRβ altered NKT cell development to an adipose-like phenotype (40) without affecting TCR activation nor its ability to bind CD1d–ligand complexes, suggesting that a hydrophobic patch created after TCRα–TCRβ pairing is essential for the development of a distinct NKT cell population (40). iNKT cells with TCRβ F108Y express E4BP4 but not PLZF, similar to AT-resident NKT cells (38). These results suggest that NKT cells in AT constitute a specialized subset and are not regular iNKT cells that localize there as passers-by.

### Mechanism of Fat Reduction *via* Thermogenesis and Relationship With Protective NKT Cells

In the development of obesity, the inflammatory environment created by NKT cell activation leads to insulin resistance and impaired glucose tolerance, which further accelerates metabolic changes that promote weight gain through increased fat mass. Meanwhile, recent studies on the suppression of obesity have provided insight into how NKT cells prevent obesity other than by producing anti-inflammatory cytokines. Fat mass is actively reduced in brown (B)AT through thermogenensis (41). BAT contains thermogenic mitochondria that express uncoupling protein (UCP) 1 and contribute to energy expenditure, in contrast to WAT (42). UCP1-expressing adipocytes with thermogenic capacity—known as beige or brite cells—also develop in WAT in response to various stimuli (43). The relationship between iNKT cells and thermogenesis was demonstrated by the finding that activated iNKT cells enhanced fibroblast growth factor 21 production and increased the number of beige cells in WAT, which in turn increased thermogenesis and weight loss (44). Several recent studies have demonstrated that innate immune cells play an important role in the induction of beige cells. vNKT cells and ILC2s induced by IL-25 produce IL-13 and regulate glucose homeostasis to protect against obesity (37). ILC2s also sustain eosinophils that produce IL-4, which stimulates M2 macrophages in VAT (45). IL-4 further stimulates M2 macrophages to secrete catecholamines for the induction of thermogenic gene expression in BAT and lipolysis in WAT (46). IL-33 is also critical for the maintenance of ILC2s in the induction of beige cells in WAT and regulation of energy expenditure. ILC2s produce methionine–enkephalin peptides that can act directly on adipocytes to upregulate UCP1 expression and promote beiging (47). These findings indicate that the innate immune system—including iNKT cells, macrophages, and ILCs—in AT controls thermogenesis by inducing beige cells, which is an important mechanism for the regulation of obesity and insulin resistance in addition to the control of AT inflammation *via* production of anti-inflammatory cytokines.

## APC for NKT Cells in AT

Natural killer T cells in DIO act as NKT1 or NKT2 (or AT-resident NKT) cells through interactions with CD1d-expressing cells in AT. Many cell types in AT express CD1d including macrophages, dendritic cells, adipocytes, and possibly others. Recent studies have shown that adipocytes can activate T cells and NKT cells through antigen presentation (48, 49). CD1d expressed on the surface of adipocytes can induce helper T cell (Th)1 and Th2 cytokine release by iNKT cells depending on the co-expression of microsomal triglyceride transfer protein and CCAAT/enhancer-binding protein-β and -δ even in the absence of exogenous ligands (48), suggesting that adipocytes express ligands that are recognized by NKT cells. To determine whether interaction between NKT cells and adipocytes influence DIO, we analyzed mice with adipocyte-specific CD1d1 deletion (adipoq^cre^-CD1d1^fl/fl^) and found that they gained less weight than control mice fed an HFD (50), consistent with our findings from conventional CD1d KO mice (24). A decrease in IFN-γ and concomitant increase in adiponectin was observed following disruption of the NKT cell/adipocyte interaction, which ameliorated AT inflammation and insulin resistance. On the contrary, another study showed that adipocyte-specific CD1d1 deletion reduced IL-4 expression in adipose iNKT cells and increased AT inflammation and insulin resistance (51), in accordance with an earlier report (49). The fact that these studies reported opposite results using the same conditional (c)KO mice provides strong evidence that adipocytes are the APCs for NKT cells in modulating AT inflammation, and that different HFDs can explain the discrepancy in the reported roles of NKT cells in the development of obesity (50, 51).

### CD1d2-Restricted NKT Cells

The fact that opposite results were obtained using the same cKO mice is critical, because it excludes the possibility that the results simply reflect the use of either pro-aggravating [CD1d1 KO; (52)] or pro-ameliorating [CD1d1/d2 KO; (53, 54)] mice (Table 1). Although it was reported that CD1d2 does not specify a specific NKT cell population (55), CD1d2 may affect the development of obesity in CD1d1 KO mice. Indeed, it was recently reported that CD1d2 can present distinct species of glycosylceramide (GlyCer) and affect the differentiation of NKT cells (56). Thus, the possible contribution of CD1d2-restricted NKT cells to the development of obesity remains to be determined, although contradictory results were obtained regarding DIO using the same cKO mice that express neither CD1d1 nor CD1d2 on adipocytes (50, 51).

**Table 1:** Roles of natural killer T (NKT) cells in the development of diet-induced obesity.

	NKT-knockout (KO)/Tg strain used	Fat source	Effect of cluster of differentiation (CD)1d deficiency or Tg on obesity	Reference
Mantell et al.	BALB/c background CD1d1/2 KO	Soybean oil, lard	No effect	(21)
Kotas et al.	B6 background CD1d1/2 KO	Soybean oil, lard	Aggravated (iNKT cell -independent effect)	(22)
Satoh et al.	B6 background CD1d1 KO	Safflower oil beef tallow	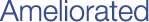 (type II NKT cells: detrimental)	(23)
Wu et al.	B6 background CD1d1 KO	Soybean oil, lard	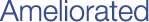 (iNKT cells: detrimental)	(24)
Lynch et al.	B6 background CD1d1/2 KO	Soybean oil, lard	Aggravated (iNKT cells: beneficial)	(34)
Ji et al.	B6 background CD1d1/2 KO	Soybean oil, lard	Aggravated (iNKT cells: beneficial)	(35)
Schipper et al.	B6 background CD1d1/2 KO	Soybean oil, lard	Aggravated (iNKT cells: beneficial)	(36)
Subramanian et al.	B6 background Vα14^Tg^/Ldlr^−/−^	High-fat, high sucrose, 0.15% cholesterol	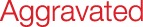 (iNKT cells: detrimental)	(31)
Satoh et al.	CD1d1^fl/fl^-*adipoq*-cre CD1d1/2 KO	Safflower oil, beef tallow	Ameliorated (iNKT cells: 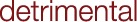 )	(50)
Huh et al.	CD1d1^fl/fl^-*adipoq*-cre CD1d1/2 KO	Soybean oil, lard	Aggravated (iNKT cells: 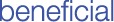 )	(51)

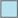
*NKT cells regulate*.

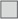
*CDld but not NKT may regulate*.

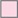
*NKT cells promote*.

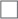
*NKT cells are neutral*.

In addition to studies of genetically engineered mice, other factors affecting the development of obesity have been investigated, including microbiota—especially those in the gut—and fat composition, both of which are related to diet and influence the presentation of ligands to NKT cells.

## Other Factors that affect the Development of DIO

### Microbiota

The findings that gut microbiota composition is a critical factor in the development of obesity come from studies using germ-free (GF) animals. Conventionally raised mice have higher total body fat than GF mice, although the latter consume more food (57). When the two types of mice are fed a sugar-rich HFD, GF mice are protected from DIO owing to increased fatty acid (FA) oxidation and AMP-activated protein kinase activity (58). On the other hand, pathogenic alterations in gut microbiome profiles (i.e., dysbiosis) in obesity affect energy metabolism (59). In fact, the abundance of *Firmicutes* is increased whereas that of *Bacteroidetes* is decreased in *ob/ob* mice with a mutation in the gene encoding leptin; on the contrary, lean *ob/*+ mice fed a polysaccharide-rich diet predominantly harbor *Bacteroidetes* (60). Similar differences in gut microbiota composition are also observed between obese and lean human subjects (61). Furthermore, GF mice inoculated with microbiota from obese twin donors developed increased adiposity when compared with those receiving transplants from lean twin donors and did not develop increased adiposity when they were cohoused with the latter mice (62). It is unclear whether microbiota or diet (calorie excess) is responsible for obesity.

Although gut microbiotas are transmissible and can be altered by diet, they may have the ability to directly alter systemic energy metabolism and thereby control weight gain. Several studies have demonstrated that NKT cells play a central role in maintaining homeostasis at mucosal surfaces (63, 64). CD1d KO mice exhibit altered gut microbiome profiles, which exacerbate intestinal inflammation induced by dextran sodium sulfate treatment and even in the steady state. Compared to non-littermate B6 mice, these mice have a higher abundance of segmented filamentous bacteria that can induce Th17 cells but reduced levels of *Akkermansia*, which may protect mice from developing colitis (65). A recent study using CD1d^fl/fl^CD11c^Cre^ cKO mice also showed that CD1d expression on CD11c^+^ cells contributes to the maintenance of intestinal homeostasis by regulation of the immunoglobulin A repertoire and induction of Tregs in the gut (66). *Bacteroides fragilis*, a prominent gut bacterial species, produces the glycosphingolipid α-GalCer_Bf_, which is structurally related to the prototypic ligand α-GalCer or KRN7000 (67). α-GalCer_Bf_ stimulates iNKT cells in the context of CD1d, suggesting that alterations in the abundance of *B. fragilis* caused by obesity can affect NKT cell homeostasis. Disruption of the NKT cell/CD1d interaction—which is required to maintain intestinal homeostasis—may affect energy consumption and fat storage by modulating gut microbiota composition.

### Fat Composition

α-Galactosylceramide is a potent activator of NKT cells, and various analogs have been synthesized that elicit distinct cytokine responses (68). For instance, α-C-GalCer and RCAI-56 promote Th1-biased responses (69, 70), whereas OCH and 20:2 promote Th2-biased responses (71). Natural ligands for CD1d have also been identified. Several mammalian glycosphingolipids such as isoglobotrihexosylceramide and β-glucosylceramide (β-GlcCer) were shown to act as self-lipid antigens (72). However, a recent study showed that a small quantity of stimulatory α-GlyCer was present in β-GlcCer preparations (73). Accordingly, pure β-GlcCer may not activate iNKT cells, which can respond to a minor fraction of α-GlyCer. Phospholipids (PHLs) that are a major component of mammalian cell membranes including phosphatidylinositol (PI), phosphatidylethanolamine (PE), and phosphatidylglycerol (PG) are natural antigens recognized by NKT cells (74). Lysophosphatidylcholine is also a natural ligand that is recognized not only by human iNKT cell clones (75) but also by Vα24^−^/Vβ11^−^ vNKT cells (76). Characteristic lipid abnormalities observed during the course of obesity include an increase in triacylglycerol and cholesterol levels in the low-density lipoprotein fraction, with a corresponding decrease in high-density lipoprotein cholesterol. In addition, obesity-related changes of serum lipids such as FAs, PHLs, and their oxidation products as well as oxylipins, sphingolipids, and their metabolites contribute to the health status and risk of comorbidities in obese patients (77). A lipidomics analysis demonstrated that changes in PHL concentrations may contribute to the development of insulin resistance and metabolic syndrome (78). Elevated circulating levels of phosphatidylcholine, PI, PE, and PG have been detected in subjects with when compared with those without non-alcoholic steatohepatitis (79). Although the molecular basis for the correlation between NKT cell activation and altered PHL levels in obese subjects remains unclear, some PHLs may affect NKT cell biology, based on the observations that the concentration of Cer species (C18:0, C20:0, and C24:1) and total Cer level was higher in type 2 diabetes; insulin sensitivity was inversely correlated with C18:0, C20:0, C24:1, C24:0, and total Cer levels; and increased tumor necrosis factor (TNF)-α concentration was correlated with the levels of C18:1 and C18:0 ceramide (80).

The mechanism of insulin resistance in obese patients with an elevated Cer concentration may involve inflammation induced by NKT cell activation, since certain Cer species stimulate NKT cells. FAs are the major components of fat and mediate immune responses. In AT, free FAs secreted by adipocytes—especially saturated FAs (SAFAs) such as palmitate and laurate—activate macrophages *via* toll-like receptor 4 to induce TNF-α expression, whereas polyunsaturated (PU)FAs such as linolenate and eicosapentaenoic acid do not have this effect (81). SAFAs, but not PUFAs, stimulate the expression of inflammatory cytokines such as IL-6 and TNF-α in adipocytes (82) that further promote metabolic syndrome. The composition and concentration of FA in sera that are altered and elevated in obese subjects are determined based on endogenous synthesis rates and dietary fat characteristics (83, 84). Thus, dietary fats may affect AT inflammation by modulating the functions of immune cells and adipocytes, suggesting that HFDs with different compositions of FA species presumably affect NKT cell response to either promote or suppress AT inflammation and obesity.

## Conclusion

**Figure 1:**
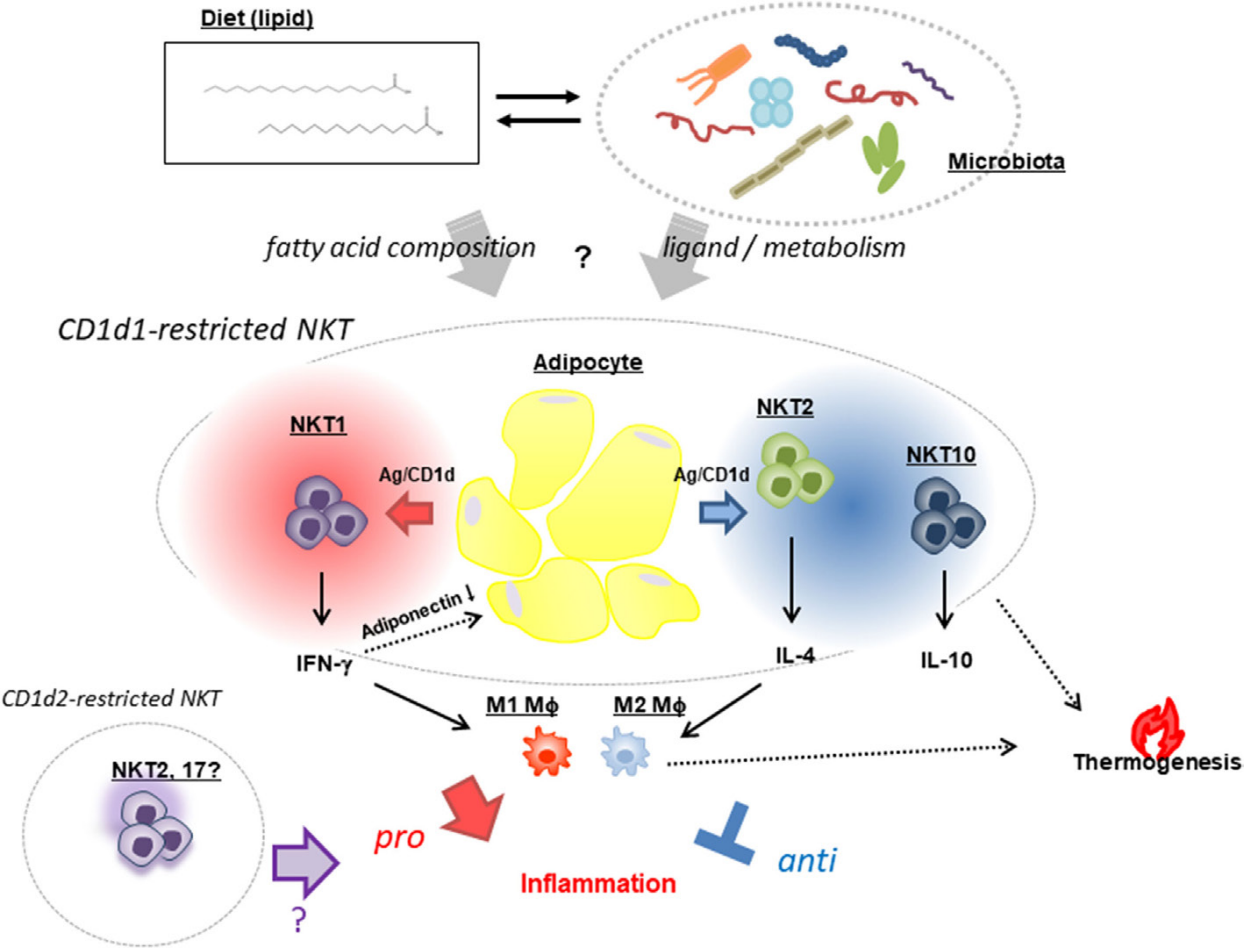
Natural killer T (NKT) cell-based modulation of AT inflammation. NKT cells exhibit opposing functions: a pro-inflammatory response that promotes AT inflammation and insulin resistance through release of IFN-γ (NKT1) that reduces the pro-ameliorating adipokine adiponectin, and a regulatory response that suppresses inflammation *via* production of IL-4 (NKT2) and -10 (NKT10) and increased thermogenesis leading to energy expenditure. These NKT [mostly cluster of differentiation (CD)1d1-restricted and very few, if any, CD1d2-restricted] cell functions are presumed to be affected by two mutually interacting factors—namely, dietary fat composition and a microbiome in which the *Bacteroidetes* and *Firmicutes* phyla predominate.

Obesity-associated inflammation in AT contributes to metabolic syndrome and is controlled by adipocytes and NKT cells with other immune cells, as discussed in this review. NKT cells appear to respond to lipid antigens on adipocytes and modulate inflammation (either by ameliorating or by aggravating this process) depending on the input—i.e., dietary lipids and ligands derived from the microbiome (Figure 1). Although the critical factors that give rise to the distinct outcomes of NKT cells remain elusive, future investigations should focus on two mutually interactive topics: (1) gut microorganisms that regulate energy consumption and modulation/maintenance by NKT cells and (2) diet/fat composition that can alter gut microbiota, the balance of lipid species, and the synthesis of endogenous lipid antigens that affect NKT cell activation. Furthermore, elucidating the mechanism of BAT maintenance and WAT beiging by NKT cells can provide a basis for the development of strategies to reverse metabolic dysregulation and reduce fat mass.

## Author Contributions

MS wrote the first draft. MS and KI edited the manuscript.

## Conflict of Interest Statement

The authors declare that the research was conducted in the absence of any commercial or financial relationships that could be construed as a potential conflict of interest.
